# World trends for *H. pylori* eradication therapy and gastric cancer prevention strategy by *H. pylori* test-and-treat

**DOI:** 10.1007/s00535-017-1407-1

**Published:** 2017-11-14

**Authors:** Hidekazu Suzuki, Hideki Mori

**Affiliations:** 1 Fellowship Training Center, Medical Education Center, Keio University School of Medicine, 35 Shinanomachi, Shinjuku-ku, Tokyo, 160-8582 Japan; 2 Department of Gastroenterology, National Hospital Organization Tokyo Medical Center, 2-5-1 Higashigaoka, Meguro-ku, Tokyo, 152-8902 Japan

**Keywords:** *H. pylori*, Gastric cancer, Clarithromycin, Metronidazole, Fluoroquinolone

## Abstract

*Helicobacter pylori*-associated gastritis leads to the development of gastric cancer. Kyoto global consensus report on *H. pylori* gastritis recommended *H. pylori* eradication therapy to prevent gastric cancer. To manage *H. pylori* infection, it is important to choose the appropriate regimen considering regional differences in resistance to clarithromycin and metronidazole. Quinolones and rifabutin-containing regimens are useful as third- and fourth-line rescue therapies.

## Introduction

*Helicobacter pylori* (*H. pylori*) is one of the main causes of gastric cancer [1, 2]. *H. pylori* leads to gastric oncogenesis through the injection of the oncoprotein CagA into host cells via a type IV secretion system [1]. Kyoto global consensus report on *H. pylori* gastritis recommended that all individuals with *H. pylori* infection should receive eradication therapy to prevent gastric cancer [3, 4]. In particular, *H. pylori* test-and-treat should be promoted in regions with high incidence of gastric cancer. Since 2013, *H. pylori* eradication therapy was approved for all cases of *H. pylori* gastritis by the national health insurance scheme in Japan, before other countries. From the global scale, a higher incidence of gastric cancer is found in Asia than in Europe and North America [5, 6]. In managing effective *H. pylori* suppression, prevalence of *H. pylori* infection and incidence of resistance to antimicrobial agents in each region are important factors. In this article, we describe the geographic characteristics of the prevalence of *H. pylori* infection, gastric cancer, and resistance to antibiotics and discuss the strategy of *H. pylori* eradication therapy.

## Epidemiology of *H. pylori* infection and gastric cancer incidence

**Fig. 1:**
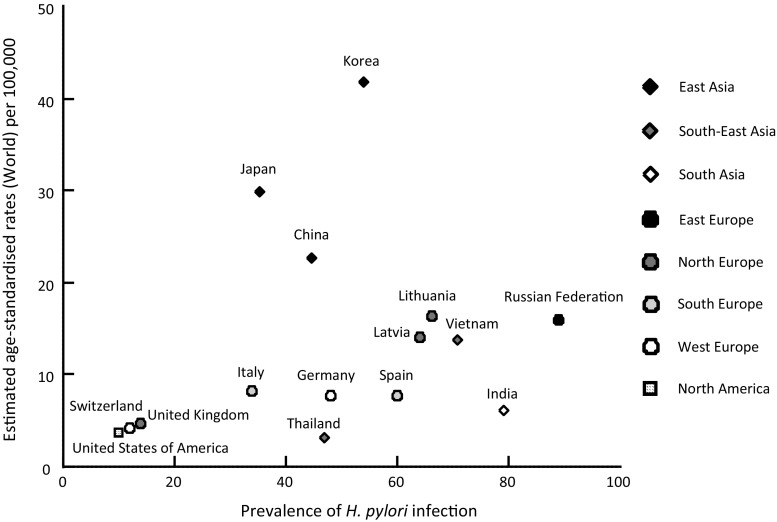
The relationship between geographic distribution of the prevalence of *H. pylori* infection and the gastric cancer incidence is described. The gastric cancer incidence is generally in direct proportion to the prevalence of *H. pylori* infection. However, higher incidences of gastric cancer are found in East Asian countries than in the other countries

The geographic distribution of the prevalence of *H. pylori* infection and gastric cancer incidence is shown in Fig. 1. The incidence of gastric cancer is generally in direct proportion to the prevalence of *H. pylori* infection [6]. However, higher incidences of gastric cancer are found in countries in eastern Asia than in other countries.

Chronic atrophic gastritis, mainly caused by *H. pylori* infection, is a known precancerous lesion for gastric cancer [7, 8]. A cross-sectional comparative study on chronic gastritis between the United Kingdom and Japan showed gastritis in Japan is histologically more severe, present at an earlier age, and more likely to be corpus predominant or pan-gastritis compared with the United Kingdom, whereas there was no significant difference in the prevalence of *H. pylori* between these countries [9].

Cytotoxin-associated gene A (CagA) is known as a major pathogenetic factor, which is peculiar to *H. pylori* [10]. To classify the genetic status of CagA, *H. pylori* CagA is classified into East Asian CagA and Western CagA [11, 12]. The East Asian CagA protein possesses stronger SHP-2 binding activity than the Western CagA [13]. The grades of inflammation, activity of gastritis, and atrophy are significantly higher in gastritis patients infected with the East Asian CagA-positive strain than in gastritis patients infected with CagA-negative or Western CagA-positive strains [12]. Therefore, the difference of CagA subtypes is the most important factor predicting the risk of gastric cancer.

There has been a progressive and rapid decline in the prevalence of *H. pylori* infection as well as a fall in the rate of progression of gastric atrophy [14], and a significant decrease in gastric cancer deaths in Japan [15]. These data indicated that a decrease in the prevalence of *H. pylori* and an increase in the attention to *H. pylori* might have a direct or indirect link to the reduction of gastric cancer deaths even in regions with high incidence of gastric cancer.

## Epidemiology of resistance to antimicrobial agents in *H. pylori*

Considering effective treatment of *H. pylori* eradication, it is important to have a deep understanding of the epidemiology of resistance to antimicrobial agents.

Resistance to amoxicillin is either null or less than 1% [16], and no significant changes in resistance were observed [17]. Therefore, amoxicillin is, and will be, a key drug in the treatment of *H. pylori*. On the contrary, we reported unsuccessful eradication treatment increased resistance even to amoxicillin [18]; thus, rescue therapy should be also determined considering amoxicillin resistance.

**Fig. 2:**
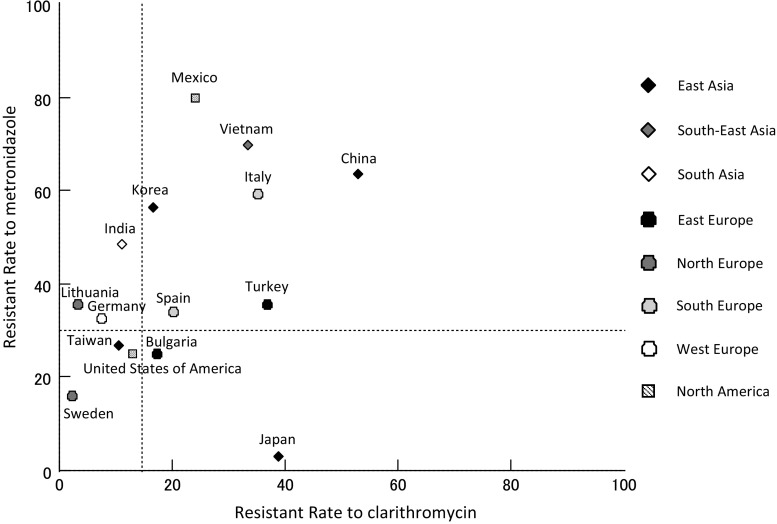
The geographic distribution of resistance to clarithromycin and metronidazole is described. There is only a limited relationship between geographic factor and the resistance to clarithromycin and metronidazole

The geographic distribution of resistance to clarithromycin and metronidazole is shown in Fig. 2 [19–34]. Interestingly, there is only a limited relationship between geographic factors and resistance to clarithromycin and metronidazole. Among the countries in eastern Asia, high resistance to clarithromycin and low resistance to metronidazole are found in Japan; low resistance to clarithromycin and high resistance to metronidazole are found in Korea; and high resistance to both clarithromycin and metronidazole is found in China. In northern Europe, there is generally a low resistance to clarithromycin. In some countries, such as Italy, China, Vietnam, and Mexico, high resistance to both clarithromycin and metronidazole is reported. A significant increase in resistance to clarithromycin and metronidazole was noted over the last year [35, 36], suggesting that acquisition of resistance is related to high consumption rates of these antibiotics.

The prevalence of primary resistance to quinolones has been reported to range from 2 to 22% [28]. The prevalence of quinolone resistance is reported to be relatively higher in Japan, Korea, and Italy (15–22%), and to be very low in China and Egypt (approximately 2%).

The prevalence of primary resistance to rifabutin, if rifamycins have not been used, is very low [37–39].

## Treatment of *H. pylori* infection

**Fig. 3:**
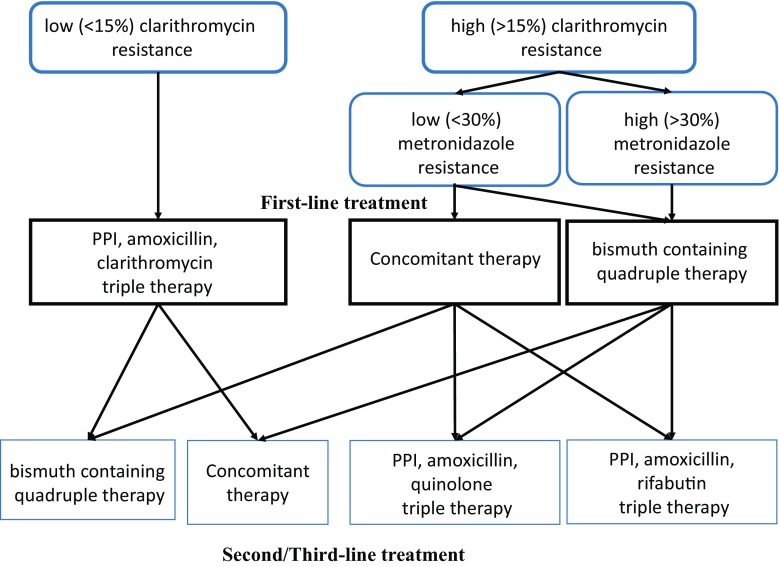
Strategy on treatment of *H. pylori* infection is shown in accordance with the Maastricht V/Florence Consensus Report [40]

A strategy for treating *H. pylori* infection is shown in Fig. 3 in accordance with the Maastricht V/Florence Consensus Report, a novel European guideline for managing *H. pylori* infection [40]. At first, the standard triple regimen of proton pump inhibitor (PPI), amoxicillin, and clarithromycin is recommended when the clarithromycin resistance rate in the region is less than 15%. Murakami et al. showed standard triple regimen (lansoprazole 30 mg bid, amoxicillin 750 mg bid, clarithromycin 400 or 800 mg bid for 7 days) achieved a successful eradication rate of 97.3% when the strains are susceptible to clarithromycin [41]. In areas of high (> 15%) clarithromycin resistance, bismuth-containing quadruple therapy is recommended as a first-line treatment. In regions with high clarithromycin resistance but low to intermediate metronidazole resistance, non-bismuth quadruple concomitant therapy (PPI, amoxicillin, clarithromycin and metronidazole) can be an alternative treatment [40, 42]. In areas of high dual clarithromycin and metronidazole resistance, bismuth-containing quadruple therapy is the recommended first-line treatment. Ten-day bismuth quadruple therapy is more effective than 10-days standard triple therapy as first-line therapy for patients with *H. pylori*-induced chronic gastritis in China (86.1 vs 58.4%, ITT analysis) [43].

After failure of the first-line treatment (triple or non-bismuth quadruple) and second-line treatment (quinolone-containing therapy), the bismuth-based quadruple therapy is recommended. Rescue therapy with bismuth-containing quadruple therapy in patients achieved 83% of successful eradications in France [44]. After failure of first-line treatment with bismuth quadruple and second-line treatment, it is recommended to use a quinolone-based triple or quadruple therapy [40]. In regions with high clarithromycin resistance but low to intermediate metronidazole resistance, triple therapy (amoxicillin, metronidazole, and PPI) is also effective when metronidazole was not used as a first-line regimen [45, 46].

Reports of third- and fourth-line treatment are described in Tables 1, 2, 3. Quinolone-based therapy is one of the most used third-line regimens. Levofloxacin have been widely used as *H. pylori* rescue therapy [47–51]. However, the effectiveness of levofloxacin-based therapies against *gyrA* mutation positive strains is insufficient; the eradication rates are approximately 40% [52, 53]. Sitafloxacin-containing treatment achieved up to 70% of successful eradications for *gyrA* mutation positive strains [54–57]. A randomized study revealed that the eradication rate of sitafloxacin-based triple regimens was better than that of levofloxacin-based triple regimens [49]. Therefore, sitafloxacin is currently the most useful fluoroquinolone, if available. However, resistance to quinolones is acquired easily, and some strains develop strong resistance via acquisition of double mutations in *gyrA* [58, 59].

**Table 1:** Reports of quinolone containing third-line regimens

Authors	Country	Publication (year)	Number of previous failed treatments	Therapy regimen	Duration	Eradication rate (%)
Zullo A et al. [47]	Italy	2003	2	RPZ 20 mg b.i.d., AMX 1 g b.i.d. and LVFX 250 mg b.i.d.	10	83
Gisbert JP et al. [48]	Spain	2006	2	OPZ 20 mg b.i.d., AMX 1 g b.i.d. and LVFX 500 mg b.i.d.	10	85
Murakami K et al. [49]	Japan	2013	2	LPZ 30 mg b.i.d., AMX 750 mg b.i.d. and LVFX 300 mg b.i.d.	7	43
Okimoto K et al. [50]	Japan	2014	2	RPZ 10 mg b.i.d., AMX 750 mg b.i.d. and LVFX 500 mg q.d.s.	10	46
Paoluzi OA et al. [51]	Italy	2015	2	EPZ 20 mg b.i.d., LVFX 500 mg b.i.d. and doxycycline 100 mg b.i.d.	7	46
Matsuzaki J et al. [54]	Japan	2012	2	RPZ 10 mg q.i.d., AMX 500 mg q.i.d. and STFX 100 mg bid for 7 days	7	84
Murakami K et al. [49]	Japan	2013	2	LPZ 30 mg b.i.d., AMX 750 mg b.i.d. and STFX 100 mg b.i.d.	7	70
Mori H et al. [55]	Japan	2016	2	EPZ 20 mg b.i.d., AMX 500 mg q.i.d. and STFX 100 mg b.i.d.	10	82
Mori H et al. [55]	Japan	2016	2	EPZ 20 mg b.i.d., MTZ 250 mg b.i.d. and STFX 100 mg b.i.d.	10	76

*RPZ* Rabeprazole, *OPZ* omeprazole, *LPZ* lansoprazole, *EPZ* esomeprazole, *AMX* amoxicillin, *LVFX* levofloxacin, *STFX* sitafloxacin, *MTZ* metronidazole

**Table 2:** Reports of rifabutin containing third- and fourth-line regimens

Authors	Country	Publication (year)	Number of previous failed treatments	Therapy regimen	Duration	Eradication rate (%)
Miehlke S et al. [60]	Germany	2006	2	EPZ 20 mg b.i.d., AMX 1 g b.i.d. and RBT 150 mg b.i.d.	7	74
Gisbert JP et al. [61]	Spain	2006	2	OPZ 20 mg b.i.d., AMX 1 g b.i.d. and RBT 150 mg b.i.d.	10	45
Gisbert JP et al. [61]	Spain	2012	3	PPIs b.i.d., AMX 1 g b.i.d. and RBT 150 mg b.i.d.	10	50
Perri F et al. [62]	Italy	2014	2	LPZ 30 mg b.i.d., AMX 1 g t.i.d. and RBT 150 mg b.i.d.	7	78
Perri F et al. [62]	Italy	2014	2	LPZ 60 mg b.i.d., AMX 1 g t.i.d. and RBT 150 mg b.i.d.	7	96
Mori H et al. [63]	Japan	2016	≥ 2	EPZ 20 mg b.i.d., AMX 500 mg q.i.d. and RBT 300 mg q.d.s.	10	83
Mori H et al. [63]	Japan	2016	≥ 2	EPZ 20 mg b.i.d., AMX 500 mg q.i.d. and RBT 300 mg q.d.s.	14	94
Ciccaglione AF et al. [64]	Italy	2016	2	PPZ 20 mg b.i.d., AMX 1 g b.i.d. and RBT 150 mg b.i.d.	10	67
Ciccaglione AF et al. [64]	Italy	2016	2	PPZ 20 mg b.i.d., AMX 1 g b.i.d., RBT 150 mg b.i.d. and bismuth subcitrate 240 mg b.i.d.	10	97

*OPZ* omeprazole, *LPZ* lansoprazole, *EPZ* esomeprazole, *PPZ* pantoprazole, *AMX* amoxicillin, *RBT* rifabutin

**Table 3:** Reports of high-dose dual therapy for third-line regimens

Authors	Country	Publication (year)	Number of previous failed treatments	Therapy regimen	Duration	Eradication rate (%)
Miehlke S et al. [60]	Germany	2006	2	OPZ 40 mg t.i.d. and AMX 1 g t.i.d.	14	70
Nishizawa T et al. [67]	Japan	2012	2	RPZ 10 mg q.i.d. and AMX 500 mg q.i.d.	14	63
Murakami K et al. [49]	Japan	2013	2	LPZ 30 mg q.i.d. and AMX 500 mg q.i.d.	14	54
Okimoto K et al. [50]	Japan	2014	2	RPZ 10 mg q.i.d. and AMX 500 mg q.i.d.	14	64

*OPZ* omeprazole, *RPZ* rabeprazole, *LPZ* lansoprazole, *AMX* amoxicillin

On the contrary, rifabutin-containing therapy has been reported as a third-line treatment [48, 60–64]. Because the resistance to rifabutin is rare, rifabutin-containing therapy can overcome *H. pylori* strains with resistance to multiple antibiotics. However, there is a concern about side effects, such as leukopenia and thrombocytopenia, and occurrence of multi-resistant strains of *Mycobacterium tuberculosis*; thus, application of rifabutin-containing therapy should be chosen very carefully [65]. Regarding duration of rifabutin-containing therapy, 10-day or longer regimens were better than 7-day regimens [63, 65]. We reported 83.3% achieved successful eradication with 10-day rifabutin-containing regimens and 94.1% with 14-day regimens [63]. Regarding PPI dosage, rifabutin therapies with high dose PPI achieved more effective eradication than normal PPI dosing [66]. Bismuth had an additional effect on rifabutin-containing regimen [64].

On the contrary, high-dose PPI and amoxicillin dual therapy is a useful option as an alternative third-line treatment regimen [49, 50, 60, 67]. We previously reported eradication rate of 63.0% with rabeprazole (10 mg qid) and amoxicillin (500 mg qid) for 14 days [67]. The eradication rates are not better than those of quinolone- or rifabutin-containing therapy; however, fewer concerns about some complications and acquisition of new drug resistance is an advantage for high-dose PPI and amoxicillin dual therapy.

Vonoprazan, a first-in-class potassium-competitive acid blocker that has a strong gastric acid secretion inhibitory effect, is spotlighted. Vonoprazan, amoxicillin, and clarithromycin triple regimen clearly improved the efficacy for eradicating clarithromycin-resistant strains when compared to the standard lansoprazole, amoxicillin, and clarithromycin triple regimen (82.0 vs. 40.0%) [41]. The reports of vonoprazan-containing regimens are limited to only the triple regimens [68]; therefore, other regimens including vonoprazan, such as bismuth-quadruple therapy or concomitant therapy, are expected.

## Effectiveness of *H. pylori* eradication for gastric cancer prevention

A large-scale prospective study showed that gastric cancer develops in persons infected with *H. pylori* but not in uninfected persons [2]; thus, it was proven that infection with *H. pylori* is the most important cause of gastric carcinogenesis. However, the effect of eradication treatment on gastric cancer risk is not well evaluated. In an open-label, randomized, controlled trial in Japan, Fukase et al. revealed *H. pylori* eradication reduced the incidence of metachronous gastric cancer even after endoscopic resection of early gastric cancer during a 3-year follow-up period [69]. Meta-analysis also showed that the occurrence of metachronous gastric cancer after endoscopic resection of early gastric cancer is significantly lower in the *H. pylori* eradication group compared with the control non-eradicated group, and that the odds ratio for the incidence of metachronous gastric cancer in the eradication group was 0.42 [70]. Systematic review and meta-analysis of randomized controlled trials, of which six individual randomized controlled trials were included, also revealed that eradication therapy reduced the risk of primary gastric cancer in healthy asymptomatic infected individuals compared with control individuals, and that the relative risk was 0.66 [71]. Take et al. showed that eradication of *H. pylori* reduced the risk of developing gastric cancer in patients with peptic ulcer diseases; however, the risk is higher in the patients with severe atrophic gastritis than those with mild or moderate atrophic gastritis [72]. These data suggested that “the earlier, the better” in *H. pylori* eradication prevents gastric cancer.

## Conclusions

*H. pylori*-associated gastritis leads to the development of gastric cancer. *H. pylori* should be eradicated in patients at a younger age, possibly after adolescence at the earliest, to prevent gastric cancer. However, even after endoscopic resection of early gastric cancer, *H. pylori* eradication reduces the incidence of metachronous gastric cancer. The incidence of gastric cancer is generally in direct proportion to the prevalence of *H. pylori* infection, even in eastern Asia where the prevalence of gastric cancer is relatively high. There are regional differences in resistance to clarithromycin, metronidazole, and quinolones. Resistance to amoxicillin and rifabutin is generally rare. To manage *H. pylori* infection, it is important to choose the appropriate regimen considering regional differences in resistance to clarithromycin and metronidazole. Quinolones or rifabutin-containing regimen are useful as third- or fourth-line rescue therapies.
